# Supplemental Nutrition Assistance Program Emergency Allotments and Food Security, Hospitalizations, and Hospital Capacity

**DOI:** 10.1001/jamanetworkopen.2023.26332

**Published:** 2023-08-09

**Authors:** Matthew Lavallee, Sandro Galea, Nadia N. Abuelezam

**Affiliations:** 1 William F. Connell School of Nursing, Boston College, Chestnut Hill, Massachusetts; 2 Boston University School of Public Health, Boston, Massachusetts

## Abstract

**Importance:**

Understanding how social policies shape health is a national priority, especially in the context of the COVID-19 pandemic.

**Objective:**

To understand the association between politically motivated changes to Nebraska’s Supplemental Nutrition Assistance Program (SNAP) policy and public health measures during the COVID-19 pandemic.

**Design, Setting, and Participants:**

This cross-sectional study used synthetic control methods to estimate the association of Nebraska’s decision to reject emergency allotments for the SNAP with food security and hospital capacity indicators. A counterfactual for Nebraska was created by weighting data from the rest of the US. State-level changes in Nebraska between March 2020 and March 2021 were included. Data were acquired from the Census Bureau’s Household Pulse Survey on individual food security and mental health indicators and from the US Centers for Disease Control and Prevention on hospital-level capacity indicators. Data analysis occurred between October 2022 and June 2023.

**Intervention:**

The rejection of additional SNAP funds for low-income households in Nebraska from August to November 2020.

**Main Outcomes and Measures:**

Food insecurity and inpatient bed use indicators (ie, inpatient beds filled, inpatient beds filled by patients with COVID-19, and inpatients with COVID-19).

**Results:**

The survey data of 1 591 006 respondents from May 2020 to November 2020 was analyzed, and 24 869 (1.56%) lived in Nebraska. Nebraska’s population was composed of proportionally more White individuals (mean [SD], 88.70% [0.29%] vs 78.28% [0.26%]; *P* < .001), fewer individuals who made more than $200 000 in 2019 (4.20% [0.45%] vs 5.22% [0.12%]; *P* < .001), and more households sized 1 to 3 (63.41% [2.29%] vs 61.13% [1.10%); *P* = .03) compared with other states. Nebraska’s rejection of additional funding for SNAP recipients was associated with increases in food insecurity (raw mean [SD] difference 1.61% [1.30%]; relative difference, 19.63%; *P* = .02), percentage of inpatient beds filled by patients with COVID-19 (raw mean [SD] difference, 0.19% [1.55%]; relative difference, 3.90%; *P* = .02), and percentage of inpatient beds filled (raw mean [SD] difference, 2.35% [1.82%]; relative difference, 4.10%; *P* = .02).

**Conclusions and Relevance:**

In this cross-sectional study, the association between social policy, food security, health, and public health resources was examined, and the rejection of emergency allotments in Nebraska was associated with increased food insecurity. Additionally, this intervention was associated with an increased rate of hospitalizations for COVID-19 and non–COVID-19 causes.

## Key Points

**Question:**

What is the association of a removal of additional Supplemental Nutrition Assistance Program funding on health and public health resources during the COVID-19 pandemic?

**Findings:**

In this cross-sectional study using synthetic control methods and survey data of 1 591 006 respondents, the rejection of additional Supplemental Nutrition Assistance Program funding to vulnerable groups was associated with increased food insecurity and hospitalizations for COVID-19 and non–COVID-19 causes.

**Meaning:**

These findings suggest that changes to social policy can have detrimental impacts on health and public health resources.

## Teaser

This cross-sectional study assesses the association of changes to Nebraska’s Supplemental Nutrition Assistance Program policy with public health measures during the COVID-19 pandemic using survey data.

## Introduction

The COVID-19 pandemic reinvigorated the US national priority of improving health equity across class and race. Food insecurity contributes to health inequity and is amenable to policy solutions. As of 2021, more than 10% of US households reported food insecurity annually,^1^ despite existing public policy interventions to address this issue. The primary policy intervention for food insecurity is the Supplemental Nutrition Assistance Program (SNAP), which in 2022 served more than 21.5 million households and distributed more than $114 billion in benefits.^2^ SNAP, which is federally funded but state administered, delivers monthly monetary allotments to households to help reduce food insecurity. These allotments are calculated using a household’s net income and the cost of US Department of Agriculture’s (USDA) Thrifty Food Plan (TFP), a food plan meant to estimate the cost of a healthy diet for low-income budgets. SNAP assumes individuals need the TFP equivalent in income for food and that individuals will spend 30% of their net income on food; the deficit is paid in SNAP allotments each month.

In the early weeks of the COVID-19 pandemic food insecurity doubled,^3^ which in part drove temporary expansions to SNAP policy. These expansions took 2 forms: (1) a 15% increase to the maximum monthly allotment for all household sizes, and (2) an increase of all household allotments to the maximum amount, which were known as emergency allotments (EA).^4,5^ While the 15% increase to maximum allotments was automatic, states have been required to apply for EA each month; states could therefore turn support on and off for their constituents. Prior work suggests these expansions reduced food insecurity in the early months of the pandemic at a national level,^6^ but there is yet to be any work on the effect of food insecurity on COVID-19 outcomes or public health resources.

Most states began applying for EA in April of 2020 and continued to apply until permanently exiting the program. Nebraska is the only state to have applied for several months of EA (March 2020 to July 2020) then declined the assistance for several months (until December 2020) then reapplied for assistance. Nebraska’s choice to reject EA was reportedly driven by return-to-normal politics,^7^ independent of any change in the rates of food insecurity or COVID-19 cases in the state.

We used these policy changes to examine the health impacts associated with policy changes during the COVID-19 pandemic. We analyzed Nebraska’s decision to not renew the EA between July and December 2020 to understand the linkage between social policy and public health. In particular, we aimed to understand the impact on food insecurity and hospital capacity indicators using synthetic control causal inference methods.

## Methods

This cross-sectional study used deidentified, publicly available data and is therefore exempt from review and informed consent per the Common Rule. The study followed the Strengthening the Reporting of Observational Studies in Epidemiology (*STROBE*) reporting guideline for cross-sectional studies.

### Data Sources

We drew from 5 public data sources. First, we used the Census Bureau’s Household Pulse Survey (HPS) and Population Estimates Program (PEP) to collect data regarding state-level food security, demographics (eg, race, gender, age), and total population size. Then, we compiled state-level data about EA from the USDA. Lastly, we used the US Centers for Disease Control and Prevention’s (CDC) COVID-19 Reported Patient Impact and Hospital Capacity by State Timeseries data set to aggregate daily reporting on public health capacity (raw hospitalization counts, open inpatient beds, and beds filled by patients with COVID-19) to a monthly level.

### Primary Outcomes

Our primary outcomes were separated into 2 main categories: food insecurity and hospital capacity indicators. We constructed 4 outcome variables across these 2 categories: rate of food insecurity, percentage of inpatient beds used by patients with COVID-19, percentage of inpatient beds used, and the percentage of inpatients who had COVID-19.

First, we created monthly rates of food insecurity by dichotomizing a 4-response question in the HPS. For food insecurity, respondents were asked about the severity of food insecurity over the last 7 days. We categorized those who reported sometimes not enough to eat and often not enough to eat as food insecure. We then used PWEIGHT, a representative weighting variable included in the HPS, to reweight the individual-level survey responses and create state-month–level means.

Second, we collected the daily rates of available public health resources and COVID-19 outcomes from the CDC’s US COVID-19 Reported Patient Impact and Hospital Capacity by State Timeseries data set and the US COVID-19 Cases and Deaths by State over Time data set. We found the mean percentage of inpatient beds used, the percentage of inpatient beds used by patients with COVID-19, and the percentage of current inpatients who had or were suspected to have COVID-19 to a monthly state level.

### Covariates and Primary Exposure

We included demographics (ie, age groups, gender, race categories), educational attainment, 2019 income brackets, household size, and people reporting to be in fair health from HPS as covariates. Race was included as a covariate because there are well-documented disparities in exposure and outcome by race. The primary exposure was Nebraska’s enrollment into the SNAP emergency allotment during the time of interest. Nebraska had applied and been approved for EA assistance from March to July 2020, issuing more than $7.5 million in assistance per month. Nebraska then did not pursue the EA funding from August to November 2020. Nebraska reextended EA assistance and again began issuing EA in December of 2020.

### Statistical Analysis

We conducted 2 statistical analyses: (1) synthetic control methods analysis where we created a synthetic Nebraska to estimate the changes associated with the intervention, and (2) a naive comparison of means in the preintervention period and the 4-month postintervention (December 2020 to March 2021). We define statistical significance as *P* < .05.

Creating a credible estimate of the association from Nebraska’s intervention required advanced methods. We created a synthetic counterfactual using synthetic control methods (SCM) to estimate the effect of Nebraska’s decision to reject EA on our primary outcomes. SCM, as developed by Abadie et al,^8,9,10,11^ offer a data-driven approach to producing synthetic counterfactuals for a unit of interest from a set of donor units.

In our setting, we created a set of synthetic counterfactuals of Nebraska (Synthetic Nebraska [SN]) for each outcome of interest using composite data from all other states (which we call donor states). The SCM constructed each SN from a weighted mean (ie, weights sum to 1) of the donor states so that the outcome of interest was as similar as possible to Nebraska in the preintervention period before the shutoff (May 2020 to July 2020). These weights were chosen by matching on the set of previously mentioned covariates, whose relative importance were decided from a data-driven algorithm (eMethods in Supplement 1). Similar to other work,^12^ we ran a set of optimization functions and chose the best performing algorithm for each SN (eMethods in Supplement 1). This synthetic counterfactual allowed us to estimate the effect associated with the policy change over the intervention period (August 2020 to November 2020).

To calculate the statistical significance of our results, we used a permutation method which iteratively assigned placebo treatments to each of the donor states and recalculated a SC producing a set of placebo estimates. We then calculated the mean squared predictive error of the preintervention period fit and the intervention period fit as a ratio (*RMSE*_Post_/*RMSE*_Pre_) for all our estimates, with *RMSE* indicating root-mean-square-error. Lastly, we used the rank of the actual ratio compared with the placebo ratios to calculate the *P* value by dividing the rank by the total number of ratios (ie, the top rank of 20 *RMSE* ratios would be 1/20 or 0.05). For all SCM calculations we used R software microsynth package, version 0.1.0 (R Foundation for Statistical Computing).

Next, because we were interested in long-term changes that may have occurred in our outcomes due to the policy change, we compared the monthly average in the preintervention period (May 2020 to July 2020) for each outcome to the monthly mean of the 4 months following the return of EA (December 2020 to March 2021). To do this we took the mean value of the outcome of interest for each period then subtracted the preintervention mean from the postintervention mean to produce a raw difference.

## Results

### Preintervention

Population demographics were compared between Nebraska and the mean of the donor states (the national mean excluding Nebraska). We analyzed survey data of 1 591 006 respondents from May 2020 to November 2020, 24 869 (1.56%) of whom lived in Nebraska. Nebraska’s population compared with other states was composed of proportionally more White individuals (88.70% [0.29%] vs 78.28% [0.26%]; *P* = .001), more male individuals (49.41% [0%] vs 48.57% [0%]; *P* < .001), and fewer Hispanic individuals (8.57% [0.92%] vs 11.09% [0.20%]; *P* < .001) (Table 1). Additionally, individuals in Nebraska were less likely to be between the ages of 51 to 65 years (24.61% [0.44%] vs 25.78% [0.22]; *P* < .001), less likely to have made more than $200 000 in 2019 (4.20% [0.45%] vs 5.22% [0.12%]; *P* < .001), and more likely to be in households sized 1 to 3 (63.41% [2.29%] vs 61.13% [1.10%]; *P* = .03) (Table 1). These differences necessitated a more rigorous counterfactual. We created synthetic counterfactuals for each outcome using SCM, which matched the covariates more closely than the national average and provided a better preintervention fit (Table 2 and the Figure). The contribution of each donor state to each SN can be viewed in eTable 1 in Supplement 1. As is standard with this method,^11^ only a handful of states make up each SN.

**Table 1.** Comparison of Population Demographics for Nebraska and the Donor States (May-November 2020)

Variable	Participants, Mean (SD)		*P* value
	National	Nebraska	
Race and ethnicity			
Asian	4.2 (0.13)	2.36 (0.54)	<.001
Black	10.91 (0.40)	3.75 (0.48)	<.001
Hispanic	11.09 (0.20)	8.57 (0.92)	<.001
White	78.28 (0.26)	88.7 (0.29)	<.001
Other	6.61 (0.34)	5.19 (0.68)	<.001
Sex			
Male	48.57 (0)	49.41 (0)	<.001
Female	51.43	50.59	
Age groups, y			
18-35	27.45 (0.87)	28.49 (1.02)	.06
36-50	25.45 (0.54)	25.39 (0.8)	.88
51-65	25.78 (0.22)	24.61 (0.44)	<.001
66-80	18.96 (0.9)	18.85 (0.86)	.83
Educational attainment			
Less than high school	1.92 (0.14)	1.62 (0.52)	.17
Some high school	4.41 (0.26)	2.74 (0.53)	<.001
High school	32.48 (0.26)	30.76 (0.68)	<.001
Some college	21.63 (0.07)	23.24 (1.09)	.002
Bachelor’s degree	16.61 (0.24)	17.97 (0.83)	.001
Graduate degree	13.35 (0.25)	12.23 (0.83)	.005
2019 Income, $			
Unreported	21.2 (7.46)	19.95 (7.68)	.76
0-24 999	11.57 (1.9)	9.7 (2.28)	.12
25 000-34 999	8.97 (1.3)	8.8 (1.39)	.82
35 000-49 999	10.17 (1.03)	11.58 (1.25)	.04
50 000-74 999	14.62 (1.47)	16.61 (2.04)	.06
75 000-99 999	11 (0.75)	12.17 (1.45)	.08
100 000-149 999	12.06 (0.79)	12.23 (1.48)	.79
150 000-199 999	5.18 (0.27)	4.77 (0.78)	.21
>200 000	5.22 (0.12)	4.20 (0.45)	<.001
Household size			
1-3	61.13 (1.10)	63.41 (2.29)	.03
4-6	32.97 (0.59)	31.48 (1.66)	.04
7-10	5.9 (1.2)	5.11 (1.49)	.29
Reporting fair health	81.37 (1.29)	83.42 (2.39)	.07

**Table 2.** Nebraska’s Emergency Allotment Policy Changes on Primary Outcomes During the 2020 COVID-19 Pandemic

Outcome	Participants, Mean (SD)^a^			Synthetic control estimate^b^	*P* value	Adjusted *P* value	Pre–post difference^c^
	National	Synthetic	Nebraska				
Food insecurity							
% Food insecure	9.78 (2.54)	8.05 (0.85)	8.20 (0.83)	1.61	.02	.03	17.68
Public health capacity							
% Of inpatient beds filled by COVID-19 patients	6.60 (5.93)	4.81 (0.72)	4.87 (0.74)	0.19	.02	.03	67.97
% Of inpatient beds filled	63.25 (9.04)	57.34 (3.64)	57.34 (3.64)	2.35	.02	.03	14.25
% Of inpatients with COVID-19	10.02 (7.8)	8.57 (1.85)	8.60 (1.87)	−0.72	.02	.03	43.84

^a^ The preintervention mean is the mean value of each outcome between May to July 2020.

^b^ The synthetic control estimate is the mean monthly change in each outcome due to the intervention. To control the false discovery rate within families of independent hypotheses, we used the Benjamini-Hochberg procedure to adjust *P* values.

^c^ The pre-post difference is the percentage point change between the preintervention mean to the mean of the 4 months after emergency allotment are reapproved (December 2020 to March 2021).

**Figure.**
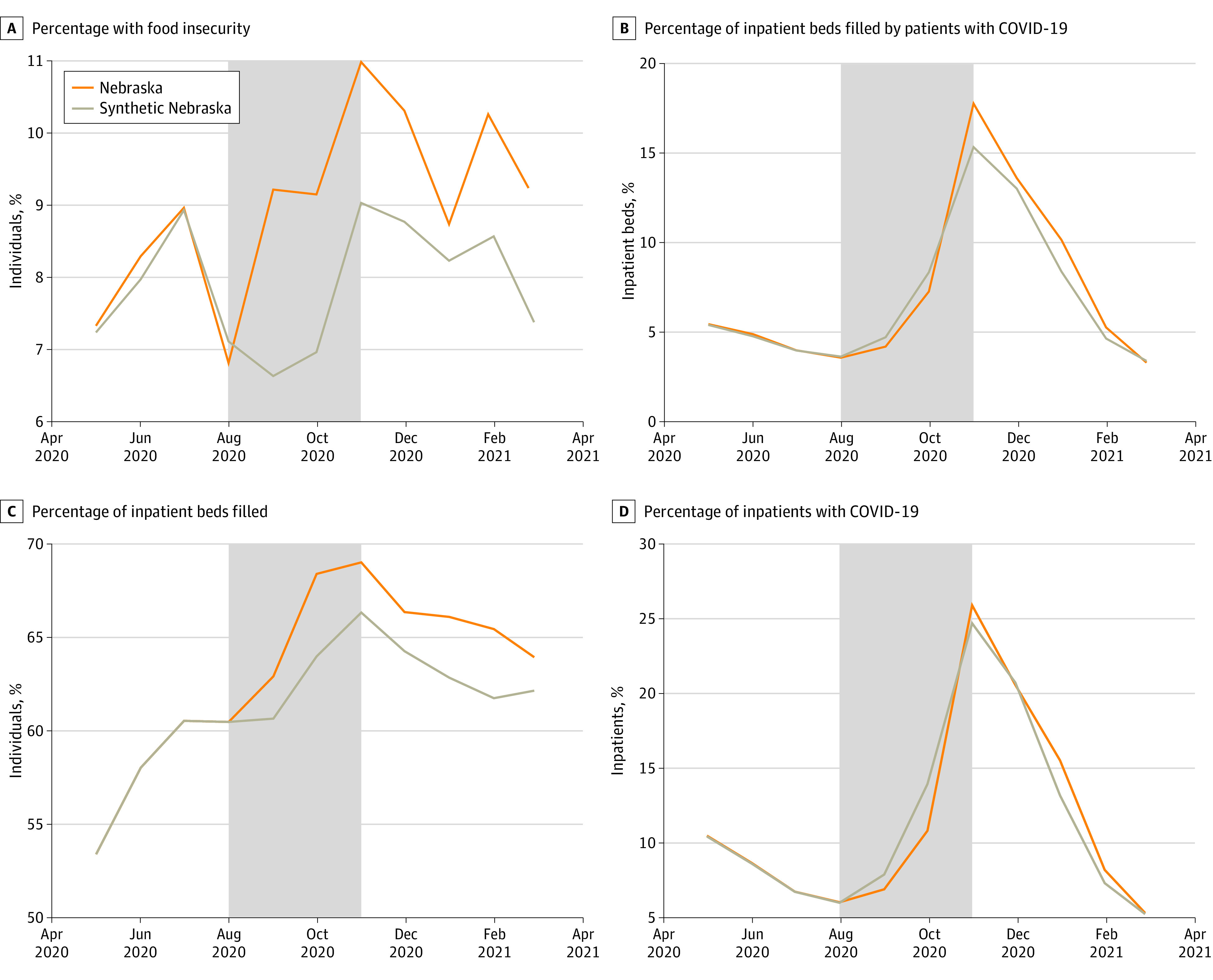
Nebraska and Synthetic Nebraska (SN) Trends in Food Insecurity, Mental Health, and Hospital Capacity Indicators

### Intervention Period

We found increases to population-level food insecurity (raw mean [SD] difference, 1.61% [1.30%]; relative difference, 19.63%; *P* = .02), percentage of inpatient beds filled by patients with COVID-19 (raw mean [SD] difference, 0.19% [1.55%]; relative difference, 3.90%; *P* = .02), and percentage of inpatient beds filled (raw mean [SD] difference, 2.35% [1.82%]; relative difference, 4.10%; *P* = .02) during the intervention period (Table 2). We also found decreases in the percentage of inpatients with COVID-19 (raw mean [SD] difference, −0.72% [1.84%]; relative difference, 8.37%; *P* = .02).

### Postintervention

We found raw increases in the postintervention period for the following outcomes: population-level food insecurity (preintervention mean, 8.20%; postintervention mean, 9.65%; relative difference, 17.68%), the percentage of inpatient beds filled by patients with COVID-19 (preintervention mean, 4.87%; postintervention mean, 8.18%; relative difference, 67.97%), the percentage of inpatient beds filled (preintervention mean, 57.34%; postintervention mean, 65.51%; relative difference, 14.25%), and percentage of inpatients with COVID-19 (preintervention mean, 8.60%; postintervention mean, 12.37%; relative difference, 43.84%) (Table 2).

### Sensitivity Analysis

To ensure our results during the intervention period were not sensitive to changes in individual months, we reported each monthly estimate individually in eTable 4 in Supplement 1. We found that, after correcting for multiple inferences (eMethods in Supplement 1), changes in individual months were not statistically significant. We analyzed a set of additional outcomes to ensure our results were not driven by a decrease in the supply of hospital beds (eTable 2 in Supplement 1).

## Discussion

Nebraska’s return-to-normal politics during the summer of 2020 initiated changes to the SNAP emergency allotment available to constituents facing food insecurity during a crucial time in the COVID-19 pandemic. In particular, we estimated that Nebraska rejected nearly $30 million in federal aid for 41 000 low-income households during this time. Our results showed a stark increase in food insecurity during and after this policy change providing evidence for a link between policy decisions and the factors that influenced health during the COVID-19 pandemic. The biological mechanism between food insecurity and COVID-19 outcomes may be explained by the connection between diet-sensitive comorbidities that increase the severity of COVID-19, like hypertension, hyperlipidemia, and diabetes.^13,14^ Nutrition is an important risk factor for COVID-19, and nutrition therapy has been recommended for standard practice with patients hospitalized with COVID-19.^15^ Therefore, changes to food insecurity are important to monitor alongside COVID-19 outcomes for vulnerable populations during the COVID-19 pandemic.

Our study found that the rejection of additional SNAP funding was associated with statistically significant changes to all hospital capacity outcomes. We found statistically significant increases in the hospital capacity measures, excluding the percentage of inpatients with COVID-19, which decreased. Similar to our findings, prior work has found that food insecurity was associated with increased inpatient hospital visits and increases in the number of days hospitalized.^16^ The fact that the percentage of individuals in inpatient care with COVID-19 dropped during our study, despite the increase in inpatient cases (COVID-19 and overall), implies that the increase associated with food insecurity was driven by non–COVID-19-related hospitalizations. As outlined above, these increases are likely driven through nutrition, but future work will be needed to understand the populations and conditions for which these effects were concentrated.

### Limitations

This study had limitations. First, the HPS did not collect data about who received SNAP during our study. Therefore, we were unable to identify whether any of our outcomes were changing for SNAP beneficiaries in particular. Second, the short preintervention period meant we had a limited period to match with the counterfactual, potentially allowing outlier months occurring in the preintervention period to impact the quality of our counterfactual. In a sensitivity analysis, we found that our fit was not sensitive to changes in the length of the preintervention period (eTable 3 in Supplement 1) . Third, the HPS relied on online responses and had a response rate of 5%; there was the potential for nonresponse bias in the HPS.^17^ We used the weights provided by HPS to address nonresponse bias to minimize the potential impact of this nonresponse on our food insecurity results. Furthermore, our study evaluates an intervention driven by local politics that could have potentially been sensitive to other policy changes occurring simultaneously. We analyzed data from early in the COVID-19 pandemic when state policies were largely driven and determined by national directives. Despite the diligence we have taken to ensure our SCM account for confounding, there may be some confounding by contextual factors we were unable to account for.

## Conclusions

In this cross-sectional study, we found that the rejection of SNAP emergency allotment was associated with increases in food insecurity and hospitalization capacity measures during the early months of the COVID-19 pandemic. Policies implemented at the state level were sensitive to both state and national politics; the SNAP expansion policy’s requirement that states opt-in on a month-by-month basis made it sensitive to politics associated with the COVID-19 response. These findings suggest that policy changes were associated with health outcomes and should be studied more closely when data are available.

## Notes

Supplement 1.
eMethods.
**eTable 1.** State Weights in Synthetic Control Models for Nebraska **eTable 2.** Association of Nebraska’s EA Policy Changes on Appendix Outcomes During the 2020 COVID-19 Pandemic **eTable 3.** Shortened Pretreatment Estimates **eTable 4.** Monthly Association of Nebraska’s EA Policy Changes on Primary Outcomes During the COVID-19 Pandemic

Supplement 2.
Data Sharing Statement

## References

[1] Coleman-Jensen A, Rabbitt MP, Gregory CA, Singh A. Household food security in the United States in 2021. Accessed June 22, 2023. www.ers.usda.gov/webdocs/publications/104656/err-309.pdf

[2] Food and Nutrition Service. SNAP data tables. US Department of Agriculture. Accessed January 19, 2023. https://www.fns.usda.gov/pd/supplemental-nutrition-assistance-program-snap

[3] America F. The impact of the coronavirus on local food insecurity in 2020 and 2021. Accessed September 8, 2022. https://www.feedingamerica.org/sites/default/files/2021-03/Local%20Projections%20Brief_3.31.2021.pdf

[4] Families First Coronavirus Response Act, HR 6201, 116th Congress (2020). Accessed July 23, 2023. https://www.congress.gov/bill/116th-congress/house-bill/6201/text

[5] Consolidated Appropriations Act, HR 133, 116th Congress (2020). Accessed July 23, 2023. https://www.congress.gov/bill/116th-congress/house-bill/133/text

[6] Bryant A, Follett L. Hunger relief: a natural experiment from additional SNAP benefits during the COVID-19 pandemic. Lancet Reg Health Am. 2022;10:100224. doi:10.1016/j.lana.2022.100224 35284905 PMC8901427

[7] Knapp F. Ricketts: not taking extra SNAP benefits sign of returning to normal. Nebraska Public Media. Accessed October 7, 2022. https://nebraskapublicmedia.org/en/news/news-articles/ricketts-not-taking-extra-snap-benefits-sign-of-returning-to-normal/

[8] Abadie A, Gardeazabal J. The economic costs of conflict: a case study of the basque country. Am Econ Rev. 2003;93(1):113-132. doi:10.1257/000282803321455188

[9] Abadie A, Diamond A, Hainmueller J. Synthetic control methods for comparative case studies: estimating the effect of California’s tobacco control program. J Am Stat Assoc. 2010;105(490):493-505. doi:10.1198/jasa.2009.ap08746

[10] Abadie A, Diamond A, Hainmueller J. Comparative politics and the synthetic control method. Am J Pol Sci. 2015;59(2):495-510. doi:10.1111/ajps.12116

[11] Abadie A. Using synthetic controls: feasibility, data requirements, and methodological aspects. J Econ Lit. 2021;59(2):391-425. doi:10.1257/jel.20191450

[12] Fuller S, Kazemian S, Algara C, Simmons DJ. Assessing the effectiveness of COVID-19 vaccine lotteries: A cross-state synthetic control methods approach. PLoS One. 2022;17(9):e0274374. doi:10.1371/journal.pone.0274374 36170293 PMC9518920

[13] Pourmotabbed A, Moradi S, Babaei A, . Food insecurity and mental health: a systematic review and meta-analysis. Public Health Nutr. 2020;23(10):1778-1790. doi:10.1017/S136898001900435X 32174292 PMC10200655

[14] Seligman HK, Laraia BA, Kushel MB. Food insecurity is associated with chronic disease among low-income NHANES participants. J Nutr. 2010;140(2):304-310. doi:10.3945/jn.109.112573 20032485 PMC2806885

[15] Fernández-Quintela A, Milton-Laskibar I, Trepiana J, . Key aspects in nutritional management of COVID-19 patients. J Clin Med. 2020;9(8):2589. doi:10.3390/jcm9082589 32785121 PMC7463687

[16] Berkowitz SA, Seligman HK, Basu S. Impact of food insecurity and SNAP participation on healthcare utilization and expenditures. University of Kentucky Center for Poverty. Accessed June 22, 2023. https://uknowledge.uky.edu/cgi/viewcontent.cgi?article=1105&context=ukcpr_papers

[17] Peterson S, Toribio N, Farber J, Hornick D. Nonresponse Bias Report for the 2020 Household Pulse Survey. United States Census Bureau. Accessed June 22, 2023. https://www2.census.gov/programs-surveys/demo/technical-documentation/hhp/2020_HPS_NR_Bias_Report-final.pdf

