# Supplementary material for: Supplemental Nutrition Assistance Program Emergency Allotments and Food Security, Hospitalizations, and Hospital Capacity
Source: JAMA Netw Open. 2023 Aug 9;6(8):e2326332. doi: 10.1001/jamanetworkopen.2023.26332 (PMC10413163; doi:10.1001/jamanetworkopen.2023.26332)
Supplement: Supplement 1. — eMethods. eTable 1. State Weights in Synthetic Control Models for Nebraska eTable 2. Association of Nebraska’s EA Policy Changes on Appendix Outcomes During the 2020 COVID-19 Pandemic eTable 3. Shortened Pretreatment Estimates eTable 4. Monthly Association of Nebraska’s EA Policy Changes on Primary Outcomes During the COVID-19 Pandemic [file jamanetwopen-e2326332-s001.pdf]

Supplemental Online Content

Lavallee M, Galea S, Abuelezam NN. Supplemental Nutrition Assistance Program emergency allotments and food security, hospitalizations, and hospital capacity. *JAMA Netw Open*. 2023;6(8):e2326332. doi:10.1001/jamanetworkopen.2023.26332

eMethods.

eTable 1. State Weights in Synthetic Control Models for Nebraska

eTable 2. Association of Nebraska's EA Policy Changes on Appendix Outcomes During the 2020 COVID-19 Pandemic

eTable 3. Shortened Pretreatment Estimates

eTable 4. Monthly Association if Nebraska's EA Policy Changes on Primary Outcomes During the COVID-19 Pandemic

This supplemental material has been provided by the authors to give readers additional information about their work.

© 2023 Lavallee M et al. *JAMA Network Open.*

eMethods

Optimization of Synthetic Control

Optimization was ran using the tidysynth R package (which draws its optimization from optimx). We run all available algorithms, which included: Nelder-Mead, BFGS, CG, L-BFGS-B, nlm, nlminb, spg, ucminf, newuoa, bobyqa, nmkb, hjkb, Rcgmin, Rvmmin.

Multiple Inference Corrections

To adjust for multiple inference, we used the Benjamini-Hochberg procedure to account for the testing of multiple outcomes in Tables 2, and 3. We examined the outcomes as one group and used the Benjamini-Hochberg procedure to control the false discovery rate at the 5% significance level. We reported adjusted P values after the unadjusted P values within the parentheses in Tables 2 and 3.

© 2023 Lavallee M et al. *JAMA Network Open.*

eTable1

eTable 1: State Weights in Synthetic Control Models for Nebraska

State	Percent food insecure	Number of inpatient beds filled per 100,000 people	Number of inpatient beds filled by COVID-19 patients per 100,000 people	Number of inpatient beds filled by COVID-19 patients per 100 cases	Number of inpatient beds per 100,000 people	Percent of inpatient beds filled	Percent of inpatient beds filled by COVID-19 patients	Percent of inpatients with COVID-19
Alabama	0%	0%	0%	0%	0%	0%	0%	0%
Alaska	0%	0%	0%	0%	0%	0%	0%	0%
Arizona	0%	0%	2%	0%	0%	3%	1%	2%
Arkansas	0%	0%	0%	0%	0%	0%	0%	0%
California	0%	0%	0%	0%	0%	0%	0%	0%
Colorado	0%	0%	0%	0%	0%	0%	2%	0%
Connecticut	0%	0%	0%	0%	0%	0%	0%	0%
Delaware	0%	0%	0%	0%	0%	0%	0%	0%
District of Columbia	4%	0%	10%	4%	1%	2%	10%	7%
Florida	0%	0%	0%	0%	0%	0%	0%	0%
Georgia	0%	0%	0%	0%	0%	0%	0%	0%
Hawaii	0%	0%	0%	0%	0%	0%	0%	0%
Idaho	0%	0%	0%	11%	21%	15%	0%	1%
Illinois	0%	0%	0%	0%	0%	0%	0%	0%
Indiana	0%	0%	0%	0%	0%	6%	0%	0%
Iowa	0%	30%	0%	3%	0%	0%	0%	16%
Kansas	0%	0%	0%	1%	0%	14%	0%	0%
Kentucky	0%	0%	0%	0%	0%	0%	0%	0%
Louisiana	0%	0%	0%	0%	0%	0%	0%	0%
Maine	23%	0%	8%	0%	0%	0%	7%	3%
Maryland	0%	0%	0%	0%	0%	0%	0%	0%
Massachusetts	0%	0%	0%	0%	0%	0%	0%	0%

© 2023 Lavallee M et al. *JAMA Network Open.*

eTable 1: State Weights in Synthetic Control Models for Nebraska

State	Percent food insecure	Number of inpatient beds filled per 100,000 people	Number of inpatient beds filled by COVID-19 patients per 100,000 people	Number of inpatient beds filled by COVID-19 patients per 100 cases	Number of inpatient beds per 100,000 people	Percent of inpatient beds filled	Percent of inpatient beds filled by COVID-19 patients	Percent of inpatients with COVID-19
Michigan	0%	0%	0%	0%	0%	0%	0%	0%
Minnesota	0%	0%	20%	32%	0%	0%	12%	11%
Mississippi	0%	0%	0%	0%	0%	0%	0%	0%
Missouri	0%	0%	0%	0%	0%	0%	0%	0%
Montana	9%	0%	0%	0%	0%	0%	0%	0%
Nevada	0%	0%	0%	0%	0%	0%	0%	0%
New Hampshire	0%	0%	0%	0%	0%	0%	0%	0%
New Jersey	0%	0%	0%	0%	0%	0%	2%	0%
New Mexico	0%	0%	0%	0%	0%	0%	0%	0%
New York	0%	0%	0%	0%	0%	0%	0%	0%
North Carolina	0%	0%	0%	0%	0%	0%	0%	0%
North Dakota	22%	22%	0%	1%	26%	20%	0%	0%
Ohio	0%	0%	0%	0%	0%	0%	0%	0%
Oklahoma	0%	0%	0%	0%	33%	0%	0%	0%
Oregon	0%	0%	0%	0%	0%	1%	0%	0%
Pennsylvania	0%	0%	0%	0%	0%	0%	0%	0%
Rhode Island	0%	0%	0%	0%	0%	0%	0%	0%
South Carolina	0%	0%	0%	0%	0%	0%	0%	0%
South Dakota	19%	0%	51%	45%	0%	0%	56%	51%
Tennessee	0%	22%	0%	0%	0%	0%	0%	0%
Texas	0%	0%	0%	0%	0%	0%	0%	0%
Utah	23%	8%	10%	2%	0%	0%	9%	10%
Vermont	0%	16%	0%	0%	16%	3%	0%	0%

© 2023 Lavallee M et al. *JAMA Network Open.*

eTable 1: State Weights in Synthetic Control Models for Nebraska								
State	Percent food insecure	Number of inpatient beds filled per 100,000 people	Number of inpatient beds filled by COVID-19 patients per 100,000 people	Number of inpatient beds filled by COVID-19 patients per 100 cases	Number of inpatient beds per 100,000 people	Percent of inpatient beds filled	Percent of inpatient beds filled by COVID-19 patients	Percent of inpatients with COVID-19
Virginia	0%	0%	0%	0%	0%	0%	0%	0%
Washington	0%	0%	0%	0%	0%	0%	0%	0%
West Virginia	0%	0%	0%	0%	3%	0%	0%	0%
Wisconsin	0%	0%	0%	0%	0%	35%	0%	0%
Wyoming	0%	0%	0%	0%	0%	0%	0%	0%

© 2023 Lavallee M et al. *JAMA Network Open.*

eTable 2

eTable 2: Association of Nebraska's EA policy changes on appendix outcomes during the 2020 COVID-19 pandemic.						
	Outcome	National mean (SD)[a]	Synthetic mean (SD)[a]	Nebraska mean (SD)[a]	Synthetic Control Estimate (P-value; Adjusted P-value)[b]	Pre-Post Difference[c]
Public health capacity						
	Number of inpatient beds filled per 100,000 people	3950.63 (1274.18)	4248.12 (112.41)	4290.47 (232.03)	353.18 (0.04; 0.06)	8.81
	Number of inpatient beds filled by COVID-19 patients per 100,000 people	419.33 (354.65)	373.15 (81.94)	377.29 (88.64)	-11.43 (0.25; 0.25)	58.93
	Number of inpatient beds per 100,000 people	6346.76 (2004.78)	7709.55 (460.99)	7718.61 (656.51)	342.82 (0.02; 0.06)	-7.74

[a]The pre-intervention mean is the average value of each outcome between May to July, 2020.

[b]The synthetic control estimate is the average monthly change in each outcome due to the intervention. To control the false discovery rate within families of independent hypotheses, we used the Benjamini-Hochberg procedure to adjust p-values.

[c]The pre-post difference is the percentage point change between the pre-intervention mean to the mean of the four months after EA are reapproved (December 2020 to March 2021).

© 2023 Lavallee M et al. *JAMA Network Open.*

eTable 3

Due to the short pre-intervention period, we tested the quality of counterfactual production process by cutting our preintervention period and estimating the remaining points. Our preintervention period was May – July, 2020, we therefore matched using May and June, and predicted July. The table below shows the difference between the realized Nebraska and synthetic Nebraska for each month and shows the P-value for the July estimate. We find that our synthetic control does not produce estimates that are statistically different for the final month of the pre-intervention period.

eTable 3: Shortened pretreatment differences.

Outcome	May[a]	June[a]	July[a]	P-value[b]
Percent food insecure	0.00	0.00	-0.47	0.55
Percent of inpatient beds filled by COVID-19 patients	0.01	0.01	-2.73	0.67
Percent of inpatient beds filled	0.00	0.00	-1.01	0.20
Percent of inpatients with COVID-19	0.01	0.01	-2.73	0.29

[a]The monthly amounts represent the difference between realized Nebraska and synthetic Nebraska.

[b]The p-value is the statistical significance of the difference between realized Nebraska and synthetic Nebraska in July.

© 2023 Lavallee M et al. *JAMA Network Open.*

eTable 4

eTable 4: Monthly association of Nebraska's EA policy changes on primary outcomes during the 2020 COVID-19 pandemic.

	Outcome	August	September	October	November
Food insecurity					
	Percent food insecure	-0.3 (0.27; P>.99)	2.6 (0.02; 0.16)	2.2 (0.02; 0.16)	1.96 (0.04; 0.2)
Public health capacity					
	Percent of inpatient beds filled by COVID-19 patients	-0.06 (0.45; P>.99)	-0.52 (0.02; 0.16)	-1.08 (0.02; 0.16)	2.44 (0.02; 0.16)
	Percent of inpatient beds filled	0 (0.94; P>.99)	2.27 (0.02; 0.16)	4.43 (0.02; 0.16)	2.7 (0.02; 0.16)
	Percent of inpatients with COVID-19	0.04 (0.33; P>.99)	-0.99 (0.02; 0.16)	-3.12 (0.02; 0.16)	1.21 (0.02; 0.16)

© 2023 Lavallee M et al. *JAMA Network Open.*
