# Supplementary material for: Supplemental Nutrition Assistance Program Emergency Allotments and Food Security, Hospitalizations, and Hospital Capacity
Source: JAMA Netw Open. 2023 Aug 9;6(8):e2326332. doi: 10.1001/jamanetworkopen.2023.26332 (PMC10413163; doi:10.1001/jamanetworkopen.2023.26332)
Supplement: Supplement 2. — Data Sharing Statement [file jamanetwopen-e2326332-s002.pdf]

Data Sharing Statement

Lavallee. Supplemental Nutrition Assistance Program Emergency Allotments and Food Security, Hospitalizations, and Hospital Capacity. *JAMA Netw Open*. Published August 09, 2023. doi:10.1001/jamanetworkopen.2023.26332

Data
Data available: No

Additional Information
Explanation for why data not available: All data used in this analysis are publicly available and can be accessed from the Census Bureau's Household Pulse Survey, Census Bureau's Population Estimates Program, Centers for Disease Control and Prevention's "COVID-19 Reported Patient Impact and Hospital Capacity by State Timeseries." Additional details about the data and the programming code for replication can be accessed at the linked GitHub repository: https://github.com/AbuelezamResearchTeam/Association-of-SNAP-and-Hospitalization.
